# Feeding Period Required by *Amblyomma aureolatum* Ticks for Transmission of *Rickettsia rickettsii* to Vertebrate Hosts

**DOI:** 10.3201/eid2009.140189

**Published:** 2014-09

**Authors:** Danilo G. Saraiva, Herbert S. Soares, João Fábio Soares, Marcelo B. Labruna

**Affiliations:** University of São Paulo, São Paulo, Brazil (D.G. Saraiva, H.S. Soares, J.F. Soares, M.B. Labruna);; Bicho do Mato Research Institute, Belo Horizonte, Brazil (D.G. Saraiva)

**Keywords:** Rickettsia rickettsii, Amblyomma aureolatum, Rocky Mountain spotted fever, vectorborne, ticks, tickborne, parasite, bacteria, Brazil

## Abstract

Rocky Mountain spotted fever is endemic to the São Paulo metropolitan area, Brazil, where the etiologic agent, *Rickettsia rickettsii,* is transmitted to humans by adult *Amblyomma aureolatum* ticks. We determined the minimal feeding period required by *A. aureolatum* nymphs and adults to transmit *R. rickettsii* to guinea pigs. Unfed nymphs and unfed adult ticks had to be attached to the host for >10 hours to transmit *R. rickettsii.* In contrast, fed ticks needed a minimum of 10 minutes of attachment to transmit *R. rickettsii* to hosts. Most confirmed infections of Rocky Mountain spotted fever in humans in the São Paulo metropolitan area have been associated with contact with domestic dogs, the main host of *A. aureolatum* adult ticks. The typical expectation that transmission of tickborne bacteria to humans as well as to dogs requires ≥2 hours of tick attachment may discourage persons from immediately removing them and result in transmission of this lethal bacterium.

## Abstract 2

As opposed to unfed ticks, transmission of *R. rickettsii* occurred in <10 minutes of attachment by adult fed ticks.

Feeding by *A. aureolatum* and Transmission of *R. rickettsii*

The tickborne bacterium *Rickettsia rickettsii* is the etiologic agent of the deadliest known rickettsiosis, Rocky Mountain spotted fever (RMSF). RMSF is referred to as Brazilian spotted fever in Brazil, where case-fatality rates are 20%–40% (*1*,*2*). The known distribution of *R. rickettsii* is restricted to the Americas, where different tick species have been implicated as vectors. The ticks *Dermacentor andersoni* and *D. variabilis* are the main vectors in the United States, and ticks of the *Amblyomma cajennense* complex are the most common vectors in Central and South America (*3*,*4*). The tick *Rhipicephalus sanguineus* has also been implicated as a vector for *R. rickettsii* in a few areas of Mexico and the state of Arizona in the United States (*5*,*6*). In the state of São Paulo, southeastern Brazil, there are 2 distinct epidemiologic scenarios of RMSF. Although *A. cajennense* is the identified vector in the countryside of the state of São Paulo (*1*,*4*), the tick *A. aureolatum* is the main vector in the metropolitan area of the city of São Paulo (*7*). A recent study on experimental infection of *A. aureolatum* with *R. rickettsii* demonstrated that the agent was preserved between life stages (transstadial maintenance) and by transovarial transmission in 100% of the *A. aureolatum* ticks for several consecutive generations; in addition, larvae, nymphs, and adults transmitted *R. rickettsii* to susceptible guinea pigs (*8*). Figure 1 illustrates an *A. aureolatum* adult tick.

**Figure 1:**
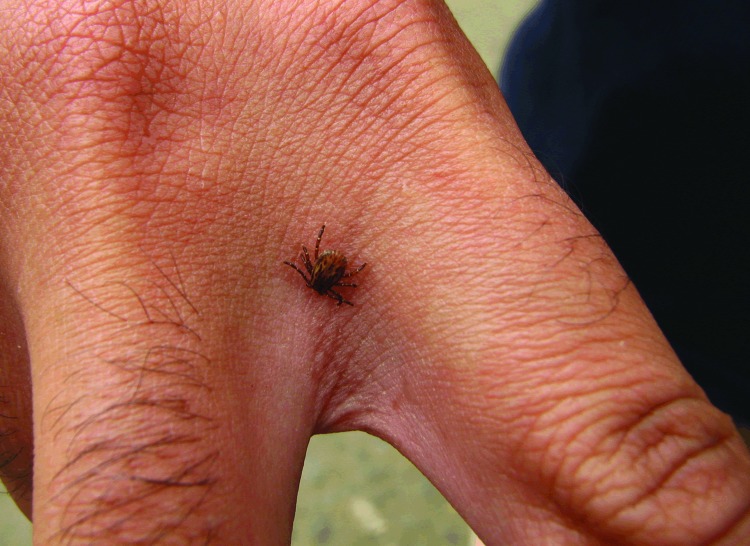
An adult male *Amblyomma aureolatum* tick attached to the hand of a person who became infested while in direct contact with a naturally infested dog in the metropolitan area of São Paulo, Brazil.

The life cycle of ticks in the hard tick family, Ixodidae, is characterized by a short parasitic phase and a long nonparasitic or free-living phase. The former consists of few days or weeks for the feeding of each of the ticks at the larval, nymphal, and adult stages; the free-living phase varies from several months to years, encompassing the off-host developmental stages (egg laying and incubation, molting), and the host-seeking period of unfed ticks (*9*). Unfed ticks are known for their capacity to survive extremely long fasting periods of months to years until they find a suitable host on which to start a new parasitic phase (*9*). During the fasting period, metabolic activity of salivary glands, midgut, reproductive organs, the excretory system, and circulation system of the tick are at much lower levels than they are during feeding periods (*9*). 

Spencer and Parker (*10*) postulated that virulence of *R. rickettsii* in tick vectors is linked directly to the physiologic state of the tick. In fasting ticks, virulent *R. rickettsii* lose their pathogenicity and virulence for guinea pigs; however, incubation of infected fasting ticks at elevated temperature (37°C) for 24 to 48 h or allowing them to feed for >48 h induces *R. rickettsii* to revert to a virulent state (reactivation). This reactivation process, or restoration of virulence, is accompanied by a series of changes in the surface structure of *R. rickettsii,* demonstrated by an ultrastructure study of the bacterium in *D. andersoni* ticks (*11*). In addition, a recent study demonstrated that the expression of some *R. rickettsii* genes is modulated by the physiologic state of the host, such as a fasting or feeding *A. aureolatum* tick (*12*); however, specific genes responsible for rickettsial reactivation remain unknown.

Earlier studies by Ricketts (*13*) and Moore (*14*) reported that adult *D. andersoni* ticks usually required a 10-hour feeding period to transmit *R. rickettsii* to vertebrate hosts, although a minimal period of 1 hour and 45 minutes was demonstrated for ticks that had previously fed on another host. Spencer and Parker (*10*) reported that this period would be >48 hours for unfed *D. andersoni* ticks. In Brazil, Magalhães (*15*) reported that *R. rickettsii*–infected *A. cajennense* adult ticks required 36 hours of feeding to transmit the agent to guinea pigs. The current literature, including medical textbooks, guidelines, and reviews on RMSF (*16*,*17*), has repeatedly advised that an infected tick requires a minimum feeding period varying from 2 to 10 hours to transmit *R. rickettsii* to humans. On the basis of this information, gathered from the above-mentioned earlier studies during the first half of the 20th century, it is widely recommended that adult persons entering wooded or grassy areas should inspect themselves and their children frequently for ticks and remove the parasites before they could efficiently transmit *R. rickettsii* (*17*).

Herein, we determined the minimal feeding period required by nymphs and adult male *A. aureolatum* ticks to transmit *R. rickettsii* to guinea pigs, since no such data have been reported for *A. aureolatum*. Male ticks were tested instead of adult female ticks because male *Amblyomma* ticks are highly motile on hosts, constantly seeking attached females (*9*). In addition, male *Amblyomma* ticks typically outnumber female ticks on hosts because male ticks can stay on hosts for a much longer period (*18*,*19*). Therefore, adult male *A. aureolatum* ticks, hereafter referred to as adult ticks, would be more likely to transmit *R. rickettsii* to humans.

## Materials and Methods

We collected 4 engorged female *A. aureolatum* ticks from dogs in São Bernardo do Campo, São Paulo metropolitan area, and brought them to the laboratory of the University of São Paulo, where we placed them in an incubator at 24°C and 95%–100% relative humidity for egg laying. We indirectly found the female offspring to be free of *Rickettsia* infection by testing the collected female ticks after oviposition by PCR, targeting a 401-bp fragment, the rickettsial *gltA* gene, as previously described (*20*). For acquisition feeding, the first generation larval progeny were allowed to feed on 5 *R. rickettsii*–infected guinea pigs previously inoculated with *R. rickettsii* strain Taiaçu, as described (*8*,*20*). This rickettsial strain had been isolated from an *A. aureolatum* tick from an RMSF–endemic area in the São Paulo metropolitan area (*21*). Recovered engorged larvae molted to nymphs; using the PCR method referenced above, we found that 10 nymphs that comprised a random sample were infected by *R. rickettsii*. Previous studies have shown that this acquisition protocol usually results in the infection of 100% of *A. aureolatum* ticks, which are capable of sustaining the rickettsial infection by transstadial maintenance and transovarial transmission (*8*,*20*).

For determination of the minimal feeding period required by an *A. aureolatum* unfed nymph to transmit *R. rickettsii* to a vertebrate host, we used 32 guinea pigs (nos. 1–32). Each guinea pig was infested by 10 *A. aureolatum* unfed nymphs, which were placed within a cotton sleeve glued to the shaved back of the animal, as described (*20*). Each of the 32 guinea pigs had a specific period in which the nymphs were allowed to feed; however, for each feeding period, we used 2 or 4 guinea pigs to replicate a given feeding period. For example, on guinea pigs 3 and 4 (Table 1), nymphs were allowed to feed for 4 hours. In this case, when the first nymph was seen attached to the skin of each animal, we started counting the feeding period. Four hours after the attachment of the first nymph, all 10 nymphs were manually removed from the guinea pig and stored frozen at −80°C until further analysis. The same procedure was used for the remaining guinea pigs, with variation of 2- to 48-hour feeding periods (Table 1). On guinea pigs 31 and 32, unfed nymphs were allowed to feed until they detached naturally as engorged nymphs, which varied from 4 to 7 days. Additional guinea pigs were infested by infected nymphs that were left to molt into adults to obtain unfed adults to be used in the following infestations.

**Table 1:** Fever, seroconversion to *Rickettsia rickettsii* antigens, and ear and/or scrotal lesions in guinea pigs exposed to *R. rickettsii-*infected *Amblyomma aureolatum* unfed nymphs through different feeding periods, Brazil

Guinea pig no.	Tick feeding period, h*	Fever†	*R. rickettsii* antibody titers‡	Ear and/or scrotal lesions§
1	2	No	<1:64	No
2	2	No	<1:64	No
3	4	No	<1:64	No
4	4	No	<1:64	No
5	6	No	<1:64	No
6	6	No	<1:64	No
7	8	No	<1:64	No
8	8	No	<1:64	No
9	8	No	<1:64	No
10	8	No	<1:64	No
11	10	No	<1:64	No
12	10	No	<1:64	No
13	12	Yes	¶	Yes
14	12	No	<1:64	No
15	12	No	<1:64	No
16	12	No	<1:64	No
17	14	Yes	2,048	Yes
18	14	No	256	No
19	16	Yes	512	No
20	16	Yes	512	No
21	18	Yes	8,192	Yes
22	18	Yes	256	No
23	24	Yes	4,096	Yes
24	24	Yes	8,192	Yes
25	24	Yes	4,096	Yes
26	24	Yes	512	No
27	36	Yes	16,384	Yes
28	36	Yes	8,192	Yes
29	48	Yes	4,096	Yes
30	48	Yes	8,192	Yes
31	>96h	Yes	16,384	Yes
32	>96h	Yes	16,384	Yes

*Number of hours that infected nymphs were allowed to feed on each guinea pig before ticks were manually removed from host. **†**Rrectal temperature >39.5°C during 21 d after tick infestation. ‡Anti-*R. rickettsii* IgG endpoint titers determined 21 d after tick infestation. §Ear or scrotal lesions (edema, necrosis) during the febrile period within 21 d after tick infestation. ¶Guinea pig died during the febrile period, before the 21st d after tick infestation; its lung was PCR-positive for *Rickettsia* spp*.*

To determine the minimal feeding period of *A. aureolatum* unfed adult ticks required to enable transmission of *R. rickettsii* to a vertebrate host, we used 24 guinea pigs (nos. 33–56). Each guinea pig was infested by 1 *A. aureolatum* unfed adult tick, as described for nymphs. Each of the 24 guinea pigs was assigned a specific feeding period in which the adult tick was allowed to feed. For example, on guinea pigs 39 and 40 (Table 2), adult ticks (1 per guinea pig) were allowed to feed for 8 hours. In this case, when the single adult tick was seen attached to the skin of each animal, we started counting the feeding period. Eight hours after attachment of the adult tick, it was manually removed from the guinea pig, and stored frozen at −80°C until further analysis. The same procedure was adopted for the remaining guinea pigs, except for the period in which the adult ticks were allowed to feed, which varied from 2 to 48 hours (Table 2). Unfed adult ticks were allowed to feed on 2 guinea pigs (nos. 55 and 56) for 7 days (168 hours), to simulate a feeding period that would last at least 7 days under natural conditions.

**Table 2:** Fever, seroconversion to *Rickettsia rickettsii* antigens, and ear and/or scrotal lesions in guinea pigs that were exposed to *R. rickettsii–*infected *Amblyomma aureolatum* unfed adult male ticks, Brazil

Guinea pig no.	Tick feeding period, h*	Fever†	Anti-*R. rickettsii* antibody titers‡	Ear and/or scrotal lesions§
33	2	No	<1:64	No
34	2	No	<1:64	No
35	4	No	<1:64	No
36	4	No	<1:64	No
37	6	No	<1:64	No
38	6	No	<1:64	No
39	8	No	<1:64	No
40	8	No	<1:64	No
41	10	No	<1:64	No
42	10	No	<1:64	No
43	12	Yes	4,096	Yes
44	12	Yes	256	No
45	16	Yes	2,048	Yes
46	16	Yes	1,024	Yes
47	20	Yes	512	Yes
48	20	Yes	2,048	Yes
49	24	Yes	2,048	Yes
50	24	Yes	¶	Yes
51	36	Yes	2,048	Yes
52	36	Yes	4,096	Yes
53	48	Yes	¶	Yes
54	48	Yes	8,192	Yes
55	168	Yes	16,384	Yes
56	168	Yes	16,384	Yes

*Number of hours that an infected male adult tick was allowed to feed on each guinea pig before the tick was manually removed from the host. **†**Rectal temperature >39.5°C during 21 days after tick infestation. ‡Anti-*R. rickettsii* IgG endpoint titers determined 21 days after tick infestation. §Occurrence of ear or scrotal lesions (edema, necrosis) during the febrile period within 21 days after tick infestation. ¶Guinea pig died during the febrile period, before the 21st day after tick infestation; its lung tissue was PCR positive for *Rickettsia* spp.

To determine the minimal feeding period required by a previously fed *A. aureolatum* adult tick to transmit *R. rickettsii* to a vertebrate host, we first allowed adult male ticks to feed with adult female ticks for 48 hours on the shaved back of tick-naïve domestic rabbits (*Oryctolagus cuniculus*), as described (*8*). Then, the fed ticks were removed from the rabbits and immediately used to infest 34 guinea pigs (nos. 57–90), as described above, except that the period in which the adult ticks were allowed to feed varied from 1 minute to 168 hours (Table 3).

**Table 3:** Fever, seroconversion to *Rickettsia rickettsii* antigens, and ear and/or scrotal lesions in guinea pigs that were infested by previously fed *R. rickettsii–*infected *Amblyomma aureolatum* adult male ticks through different feeding periods, Brazil

Guinea pig number	Tick feeding period*	Fever†	Anti-*R. rickettsii* antibody titers‡	Ear and/or scrotal lesions§
57	1 min	No	<1:64	No
58	1 min	No	<1:64	No
59	3 min	No	<1:64	No
60	3 min	No	<1:64	No
61	5 min	No	<1:64	No
62	5 min	No	<1:64	No
63	10 min	No	<1:64	No
64	10 min	Yes	1,024	No
65	20 min	Yes	1,024	No
66	20 min	Yes	512	No
67	40 min	Yes	1,024	No
68	40 min	Yes	4,096	Yes
69	1 h	Yes	4,096	Yes
70	1 h	Yes	8,192	Yes
71	2 h	Yes	¶	Yes
72	2 h	Yes	512	No
73	4 h	Yes	¶	Yes
74	4 h	Yes	16,384	Yes
75	6 h	Yes	¶	Yes
76	6 h	Yes	¶	Yes
77	8 h	Yes	¶	Yes
78	8 h	Yes	¶	Yes
79	12 h	Yes	¶	Yes
80	12 h	Yes	¶	Yes
81	18 h	Yes	¶	Yes
82	18 h	Yes	8,192	Yes
83	24 h	Yes	8,192	Yes
84	24 h	Yes	16,384	No
85	36 h	Yes	¶	Yes
86	36 h	Yes	¶	Yes
87	48 h	Yes	¶	Yes
88	48 h	Yes	¶	Yes
89	168 h	Yes	16,384	Yes
90	168 h	Yes	¶	Yes

*Number minutes or hours that an infected adult male tick was allowed to feed on each guinea pig before the tick was manually removed from the host. All ticks had previously fed on rabbits for 48 h.

†Rectal temperature >39.5°C during 21 days after tick infestation.

‡ Anti-R. rickettsii IgG endpoint titers determined at 21 days after tick infestation.

§ Ear or scrotal lesions (edema, necrosis) during the febrile period within 21 days after tick infestation.

¶Guinea pig died during the febrile period, before day 21 after tick infestation; its lung was PCR positive for Rickettsia spp.

Every guinea pig or rabbit used in this study was tick naive; these animals were provided by a private laboratory that raised the animals under proper sanitary conditions. The rectal temperatures of guinea pigs and rabbits were measured daily from the day of infestation through 21 days afterward. These animals were considered febrile if rectal temperature reached values >39.5°C (guinea pigs) or >40°C (rabbits). All animals were tested for seroconversion to *R. rickettsii* antigens. For this purpose, we collected blood samples at 0 and 21 days postinfestation and tested for anti–*R. rickettsii* reactive antibodies by immunofluorescence assay, as described (*8*,*22*). Animals were considered seronegative if their serum was not reactive at the 1:64 dilution. Some infested guinea pigs that died before day 21 postinfestation were not tested by immunofluorescence assay because a second blood sample was not obtained; however, we submitted a fragment of their lung tissue to DNA extraction using the DNeasy Tissue Kit (QIAGEN, Chatsworth, CA, USA) and tested the samples by the same PCR protocol referenced above. Clinical alterations, such as ear or scrotal necrosis, were noted when observed. We tested all nymphal and adult ticks that were manually removed from the infested guinea pigs individually by the same PCR protocol referenced above.

## Results

All PCRs performed on the DNA of nymphal and adult ticks that fed on guinea pigs for different periods resulted in amplicons compatible with *R. rickettsii,* indicating that all 90 guinea pigs in this study were exposed to *R. rickettsii–*infected ticks. Among guinea pigs exposed to *R. rickettsii*–infected unfed nymphs, animals remained afebrile and seronegative when nymphs fed for ≤10 hours (Table 1). When nymphs fed for 12 hours on 4 guinea pigs, 3 of these animals (nos. 14–16) remained seronegative and afebrile, but the fourth animal (no. 13) became febrile and died on the second week, when ear and scrotal necrosis were evident. Its lung tissue sample was PCR–positive for rickettsiae*.* All guinea pigs on which nymphs fed for 14 to ≥96 hours seroconverted to *R. rickettsii,* and fever developed in all but 1 (no. 18). Of 16 these animals, 5 did not show ear or scrotal lesions.

Among the 24 guinea pigs exposed to *R. rickettsii*–infected unfed adult ticks, 10 animals remained afebrile and seronegative when ticks fed for <10 hours (Table 2). Fever developed in the 14 guinea pigs on which the male ticks fed for 12–168 hours. Seroconversion to *R. rickettsii* was demonstrated in 12 of the 14 febrile guinea pigs. Two guinea pigs, nos. 50 and 53, died before 21 days; their lung tissue specimens were PCR–positive for rickettsiae. Thirteen of these febrile animals showed ear and scrotal lesions.

Of the 2 rabbits on which adult *A. aureolatum* ticks fed for 48 hours, 1 became febrile at day 5 and the other at day 7 postinfestation; ear necrosis developed in both rabbits, and blood samples seroconverted to *R. rickettsii* with endpoint titers of 8,192 or 16,384. When exposed to the *R. rickettsii*–infected adult ticks that had fed for 48 hours on rabbits, guinea pigs remained afebrile and seronegative when the ticks fed for ≤5 minutes (Table 3). Of 2 guinea pigs on which adult ticks fed for 10 minutes (nos. 63 and 64), no. 63 remained seronegative and afebrile, but no. 64 became febrile and seroconverted. Fever developed in all 26 guinea pigs on which fed adult ticks fed for 20 minutes to 168 hours; of these, 21 had ear or scrotal lesions, or both. Thirteen animals seroconverted to *R. rickettsii*, and 14 died during the febrile period; their lungs were positive for *Rickettsia* spp. by PCR*.*

The infection with *R. rickettsii* in guinea pigs was confirmed by seroconversion (nonfatal cases) or by PCR on lung tissue (fatal cases). Fever onset was registered between 5 and 9 days (mean 6.8) postinfestation with nymphs, between 4 and 8 days (mean 5.6) postinfestation with infected unfed adult ticks, and between 4 and 11 days (mean 6.7) postinfestation with prefed adult ticks. Among the 17 guinea pigs that became infected by *R. rickettsii* after being exposed to unfed nymphs, only 1 died of spotted fever (6% fatality rate). Among the 14 guinea pigs that became infected after being exposed to unfed adult ticks, 2 (14% fatality rate) died of spotted fever. When guinea pigs were exposed to infected ticks that had previously fed on rabbits (prefed adult ticks), the fatality rate rose to 52% (14/27).

## Discussion

This work showed that unfed nymphs and unfed adult male ticks of *A. aureolatum* needed to be attached for >10 hours on the host, to successfully transmit a virulent strain of *R. rickettsii.* In contrast, fed adults needed only up to 10 minutes of attachment for transmission of *R. rickettsii* to the host. The >10-hour feeding period observed for unfed ticks is similar to the 10-hour period previously reported for *D. andersoni* ticks in 2 earlier studies (*13*,*14*); albeit much lower than the periods previously reported for *D. andersoni* (>48 hours) in another study (*10*) and for *A. cajennense* ticks (36 hours) in Brazil (*15*). Regarding fed ticks, the 10-minuteperiod herein observed for *A. aureolatum* ticks is much shorter than the 1 hour and 45 minutes previously reported for prefed *D. andersoni* ticks (*14*). It is possible that different tick species require different feeding periods for effective inoculation of *R. rickettsii* into the host; however, it is clear that prefed ticks require much shorter periods than unfed ticks. This difference should be related to the reactivation phenomenon; i.e., *R. rickettsii* was in a nonvirulent state in unfed nymphal and adult *A. aureolatum* ticks and in its virulent state (reactivated) in the prefed adult ticks used to infest guinea pigs.

**Figure 2:**
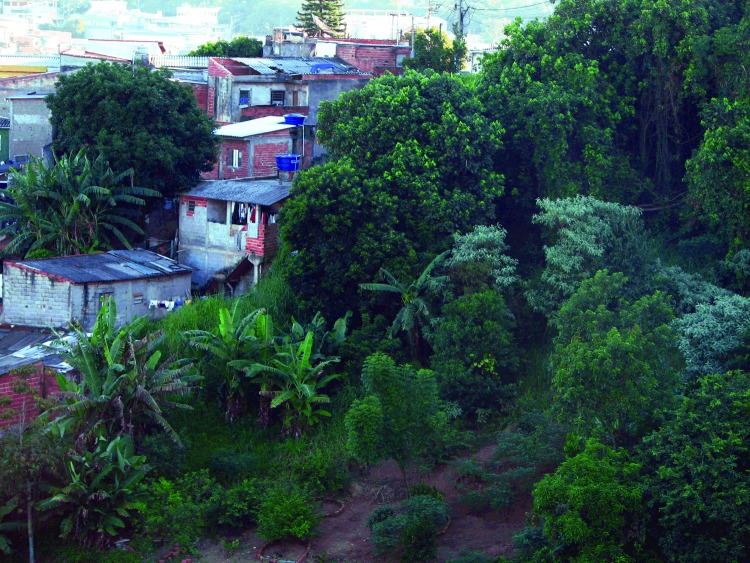
A typical area where infection with the *Rickettsia rickettsii* bacterium occurs, manifested as Rocky Mountain spotted fever, in the metropolitan area of São Paulo, Brazil. Humans have constructed their homes in the Atlantic rainforest fragment (habitat of the *Amblyomma aureolatum* tick, a vector of *R. rickettsii*), where many dogs are unrestrained. Dogs frequently enter the forest, become infested by adult *A.aureolatum* ticks, and bring them into homes, allowing the direct transfer of feeding ticks from dogs to humans.

Adult *A. aureolatum* ticks feed chiefly on *Carnivora* species (mostly domestic dogs), but immature ticks (larvae, nymphs) generally feed on passerine birds and a few rodent species (*7*,*23*). Humans have reported being attacked only by adult ticks, and usually by a single tick (*24*), because the population density of *A. aureolatum* ticks is usually low (*18*). In southeastern Brazil, the distribution of *A. aureolatum* populations is restricted to Atlantic rainforest fragments where optimal conditions of high humidity and cool temperatures prevail throughout the year (*7*,*18*). Therefore, infestations occur typically on domestic dogs that are reared unrestrained, with access to Atlantic rainforest fragments (*18*,*25*). However, to our knowledge, *A. aureolatum–*human infestation acquired in the forest has not been studied and documented. In fact, in an Atlantic rainforest reserve in the state of São Paulo, 4 *Amblyomma* tick species (including *A. aureolatum*) were collected in wild animal trails during a 4-year period (*26*), when *A. aureolatum* was the only 1 of the 4 tick species that was not reported to have attached to researchers during their field activities in the forest (*27*). Thus, we hypothesize that many of the RMSF-confirmed cases in the São Paulo metropolitan area were transmitted by *A. aureolatum* ticks that had fed on domestic dogs. In this case, the domestic dog would have become infested in the forest and brought an infected tick indoors, where it came into direct contact with humans (Figure 2). This statement is corroborated by a study that reported that 69% of the RMSF cases in the São Paulo metropolitan area occurred in children and women, who usually did not enter the forest (habitat of *A. aureolatum*) as frequently as did adult men (*28*). In addition, 93% of the cases in this area have been associated with direct contact with dogs (*29*).

In this study, the fatality rate for guinea pigs exposed to prefed adult ticks (52%) was much higher than the rate for guinea pigs exposed to unfed ticks (14%). A recent study reported that the fatality rate for patients with RMSF in a region of the São Paulo metropolitan area (transmission by *A. aureolatum* ticks) was 62.5% during 2003–2010, which was substantially higher than the 33.3% fatality rate observed in a region of the countryside of the state of São Paulo (transmission by *A. cajennense* ticks) during a similar period (*29*). Similarly to the situation with the guinea pigs in this study, this marked difference among RMSF case-patients could be related to the reactivation state of *R. rickettsii* in the tick vector, since we postulated above that infestation by fed ticks would predominate in the metropolitan area of São Paulo. In the countryside, acquisition of *R. rickettsii* infection could be predominantly related to infestations by unfed *A. cajennense* ticks acquired directly in the field, since such infestations are commonly reported in this area (*4*,*30*,*31*).

According to results of this study, a fed *A. aureolatum* tick could transmit *R. rickettsii* to a human in as few as 10 minutes of parasitism. Because this route of transmission seems to be common in the metropolitan area of São Paulo, health authorities must be aware that current textbooks and guidelines that indicate that an infected tick takes 2 to 10 hours to transmit *R. rickettsii* to humans (*16*,*17*) do not apply to the São Paulo metropolitan area.

In the eastern United States, *R. rickettsii* is transmitted to humans typically by the *D. variabilis* tick in the adult stage, commonly known as the American dog tick, which feeds chiefly on domestic dogs (*17*). Similarly to the circumstances in the São Paulo metropolitan area, most of the RMSF cases in the eastern United States have occurred in children and women (*32*,*33*), and infections in canines have been associated repeatedly with an increased risk for disease in owners (*34*). Because numerous reports of infected humans were associated with tick-infested dogs or tick removal within 4 weeks of disease onset, researchers have proposed that many of these cases were a result of direct contact with rickettsiae from tick body fluids during tick removal (*34*,*35*). Although this postulated mechanism cannot be discarded (including in the São Paulo metropolitan area), the current literature has considered that an attached tick needs several to many hours of attachment for a successful inoculation of rickettsiae into human skin. Once it is forcibly removed from a host, a partially fed tick loses its discriminatory senses and strives to feed wherever possible on any available vertebrate animal (*36*). Thus, it is reasonable to consider that tick removal habits in RMSF-endemic areas could have implications for the transmission of *R. rickettsii,* not only caused by potential direct contact with tick fluids, but also, as shown in this study, because detached ticks could readily attach to humans and inoculate them with rickettsiae within few minutes.
